# Categorical and continuous - disentangling the neural correlates of the carry effect in multi-digit addition

**DOI:** 10.1186/1744-9081-6-70

**Published:** 2010-11-20

**Authors:** Elise Klein, Klaus Willmes, Katharina Dressel, Frank Domahs, Guilherme Wood, Hans-Christoph Nuerk, Korbinian Moeller

**Affiliations:** 1 Section Neuropsychology, Department of Neurology, University Hospital, RWTH Aachen University, Germany; 2 Interdisciplinary Center for Clinical Research Aachen, RWTH Aachen, Germany; 3 Department of Psychology, Eberhard Karls University, Tuebingen, Germany; 4 Department of Psychology and Center of Neurocognitive Research, Paris-Lodron University of Salzburg, Austria; 5 IWM-KMRC Knowledge Media Research Center, Germany

## Abstract

**Background:**

Recently it was suggested that the carry effect observed in addition involves both categorical and continuous processing characteristics.

**Methods:**

In the present study, we aimed at identifying the specific neural correlates associated with processing either categorical or continuous aspects of the carry effect in an fMRI study on multi-digit addition.

**Results:**

In line with our expectations, we observed two distinct parts of the fronto-parietal network subserving numerical cognition to be associated with either one of these two characteristics. On the one hand, the categorical aspect of the carry effect was associated with left-hemispheric language areas and the basal ganglia probably reflecting increased demands on procedural and problem solving processes. Complementarily, the continuous aspect of the carry effect was associated with increased intraparietal activation indicating increasing demands on magnitude processing as well as place-value integration with increasing unit sum.

**Conclusions:**

In summary, the findings suggest representations and processes underlying the carry effect in multi-digit addition to be more complex and interactive than assumed previously.

## Introduction

Previous studies indicated that addition performance depends strongly on whether or not a carry operation is needed to solve the task. Whenever a carry operation is required, response latencies, error rates as well as functional brain activation increase considerably [1-6]. Generally, the need for a carry operation is determined by the summands of the addition problem: whenever the sum of the unit digits in one corresponding place position of an addition problem becomes equal or larger than 10 a carry operation is necessary to compute the correct result (e.g., 8 + 6 > 10 in 38 + 26). In contrast, no carry operation is needed whenever the sum remains smaller than 10 (e.g., 2 + 3 < 10 in 52 + 23). In the above example 38 + 26, the carry operation is executed by adding 1 (i.e., *carrying *the decade digit of the unit sum from the unit to the decade position) to the sum of the decade digits of the summands (i.e., 3 + 2 + 1 = 6) - thereby updating the decade digit of the result by the so-called carry.

At the behavioral level, increased response latencies and error rates were observed repeatedly to reflect increased difficulty of carry addition problems [1-3,7]. Moreover, when evaluating the neuro-functional correlates of increased difficulty of carry problems, an additional recruitment of (pre)frontal cortices was observed (e.g., [6]). However, activation within these areas does not necessarily seem specific for number magnitude processing itself (cf. [8]). Instead, this activation may rather reflect more general processes involved in complex calculation. These processes may comprise (i) increased working memory demands (e.g., [2-4,9,10]); (ii) the resolution of interference and processes of response selection (e.g., [11]); (iii) the processing of additional operations in calculation (e.g., [12]); (iv) processes of cognitive control (e.g., [13]). Taken together, it is an established finding that carry addition problems are more difficult than non-carry problems. In line with this rationale the carry effect is usually assumed to be categorical, meaning that there is either the need for a carry operation posing higher demands on, for instance, working memory, knowledge of arithmetic procedures, cognitive control, etc. or not. However, recent research indicated that such a categorical view might only be part of the story.

### The nature of the carry effect

Only recently, Klein et al. [14] suggested that the carry effect might not only be of a purely categorical nature; rather, it may involve continuous properties as well. In particular, it was observed that both reaction time and error rates increased commensurately with the sum of the unit digits (i.e., 24 + 37 is easier than 17 + 39 because of 4 + 7 = 11 < 7 + 9 = 16 even though both problems require a carry operation and sum up to an approximately similar problem size). The idea of decomposed processing of the magnitude of the constituent unit and decade digits (see [15], for a review) suggests the carry effect to be driven at least in part by specifically processing the magnitudes of the unit digits of the summands. In this vein, the Klein et al. study [14] aimed at differentiating the influence of decade and unit digits of the summands. More particularly, they compared the influence of the unit digits of the summands to a categorical carry predictor in a regression analysis by incorporating both of them in the regression analysis. The decomposed processing of tens and units reflected by the use of two predictors indexing either tens or unit influences is actually in line with the calculation procedure for addition as taught at school: column-wise processing from right to left. In any case, it is the sum of the single-digits in one column that determines, whether a carry to the next column to the left is needed or not (in the present case of two-digit addition unit sum ≥ 10 vs. unit sum < 10). When running a step-wise regression analysis with the above mentioned predictors, Klein et al. [14] found unit sum to be a significant predictor of item RT, whereas the categorical carry predictor was not considered for the final model. However, it is important to note that the categorical carry predictor and the continuous predictor unit sum are highly correlated (r = .86, for the stimulus set employed). Therefore, Klein et al. [14] refrained from claiming that the carry effect may be either categorical or continuous in nature. Instead, the authors suggested that the carry effect indeed involves both categorical and continuous aspects. Moreover, only behavioural data was reported. Therefore, Klein and colleagues [14] were only able to draw a preliminary conclusion with respect to what these categorical and continuous aspects might indicate on a more procedural and associated theoretical level. The authors suggested that, on the one hand, influences captured by the categorical predictor might reflect processes associated with the additional demands on cognitive control and/or working memory only for a carry operation. On the other hand, influences reflected by the continuous carry predictor (i.e., unit sum) may rather be associated with specific processing of numerical information such as the magnitude of the involved digits and/or processes of place-value integration.

Considering the specific processing characteristics implied by either the categorical or the continuous carry predictor, one should be able to evaluate their differential origin by means of identifying their neural correlates. As already described for the case of the categorical processing aspect, increased demands on cognitive control, (verbal) working memory, interference resolution, etc. should be primarily reflected by the involvement of (pre)frontal cortices in carry addition problems [6,11-13]. On the other hand, continuous characteristics may indeed represent a special case of (column-wise) manipulation of numerical magnitude. Based on the latter argument, the continuous aspect of the carry effect should primarily be associated with magnitude-related activation. Previously, processing number magnitude (for a review see [16]) as well as place-value integration were both associated specifically with activation of the intraparietal sulcus (IPS; see [17,18], see also [19] for a distinction between the neural correlates of processing single- and two-digit numbers). First neuro-functional evidence for the continuous aspects comes from an fMRI study by Klein and co-workers [5]. The authors observed that the requirement of a carry did not result in an increase of frontal activation; instead, the most pronounced change of the fMRI signal was found within the IPS (see also [12] for increases in IPS activation with numerical task complexity). In summary, the carry from one position (e.g., units) to the next higher power of ten (e.g., tens) within the place-value structure of the Arabic number system may be associated with both categorical *and *continuous characteristics. Categorical differences between carry and non-carry problems may tap on working memory, cognitive control, etc, while continuous characteristics may imply a special case of number magnitude processing. The current study pursued this issue.

Finally, it should be noted that not only the requirement of a carry operation influences addition performance. Previous studies also indicated that addition performance is determined by the magnitude of the addends involved (the so-called problem size) and attributes of distractors used in the case of verification/choice reaction paradigms [14,21]. Therefore, in order to evaluate the nature of the carry effect properly, it is necessary to take into account these known factors.

## Objectives

The current study was set up to investigate the nature of the carry effect and its neural correlates systematically. Applying a parametric analysis to the functional MRI data, we aimed at replicating the so far purely behavioural finding of both categorical and continuous aspects of the carry effect at the neural level. In particular, we hypothesized to find two distinct parts of a neural network associated with each of these two aspects: on the one hand, we expected to find one part of the network reflecting the categorical aspect of the carry effect, possibly subserved by (pre)frontal cortices assumed to host processes of cognitive control and (verbal) working memory. On the other hand, the continuous aspects of the carry effect we expected to be associated with a different, magnitude-related part of the network primarily involving the IPS.

## Methods

### Participants

17 male right-handed volunteers (mean age = 28 years; SD = 5) participated in the study after having given their written informed consent in accord with the protocol of the local Ethics Committee of the Medical Faculty of the RWTH Aachen University.

### Stimuli

A total of 96 different single- and two-digit addition problems in Arabic notation were employed In a choice-reaction paradigm. Each addition problem was presented centrally above a pair of solution probes. Participants had to indicate the correct result by pressing a corresponding button with either the left or the right hand. The experimental within-participant 2 × 2 × 2 design comprised the three factors carry (carry vs. non-carry; e.g., 27 + 48 vs. 21 + 48), problem size (sum < 40 vs. > 60; e.g., 13 + 14 vs. 13 + 54), and distractor type. Half of the incorrect solution probes (distractors) differed from the correct result by ±2, whereas the distance between the correct result and the distractor was ±10 for the other half, in order to minimize parity based solution strategies [21-23]. Additionally, half of the distractors differed from the correct result only in the units position, while in the other half only the tens position was different from the correct result. Thus, trying to identify the distractor by focusing on either tens or units would not be a beneficial strategy.

Finally, the following stimulus properties were matched between the respective stimulus categories carry vs. no carry: absolute sum (equalling problem size), logarithmic sum, mean magnitude of the unit, mean magnitude of the decade digit of the correct result and the distractor. On the other hand, the need for a carry was matched between problems with either small or large problems. Generally, the position (left/right) of the smaller addend within the problem, the occurrence of the digit 5 at either the units or tens position of the addends as well as the correct result/distractor, and the parity of the correct result and the distractor were held constant between all four stimulus categories (i.e., small problem size no carry, small problem size carry, large problem size no carry, large problem size carry). Klein et al. [14] provide a list of all stimuli used including their properties. Neither ties nor multiples of ten were included as either addends or probes. Additionally, the unit digits of the addends were different in all problems. Moreover, no addition problem was part of a multiplication table (e.g., 16 + 24).

### Procedure

The experiment was a combined rapid event-related fMRI and reaction time (RT) study. Video goggles designed to meet MR requirements [24] simulated a distance of about 1.20 m from a monitor. All stimuli were in white Arial 100 font against a black background using Presentation software [25]. At these settings, single digits were displayed with height and width being about 2.0° and 1.1° of visual angle, respectively. Head movements were restrained by soft foam pads and a head coil.

Participants were instructed to indicate as fast and as accurate as possible, which one of the two solution probes was the correct result, by pressing a corresponding response button. To familiarize participants with task requirements and to reduce training effects during fMRI acquisition, participants had to solve 36 addition problems before being examined in the scanner. None of these items was part of the original experiment. All 96 trials were presented successively in one run, lasting about 10 minutes. Each addition problem was displayed for 4.5 seconds even when a response was given before the end of the presentation period. Trial order was pseudo-randomized with each participant performing the same sequence of trials. Each addition problem appeared only once.

### Scanning procedure and data acquisition

#### MRI acquisition

For each participant, a high-resolution T1-weighted anatomical scan was acquired with the Philips 1.5T Gyroscan MRI system using a standard head coil (TR = 30 ms, matrix = 256 × 256 mm, 160 slices, voxel size = 1 × 1 × 1 mm; FOV = 256 mm, TE = 4.6 ms; flip angle = 30°).

#### fMRI acquisition

One functional imaging run sensitive to blood oxygenation level-dependent (BOLD) contrast was recorded for each participant (T2*-weighted echo-planar sequence, TR = 2800 ms; TE = 50 ms; flip angle = 90°; FOV = 240 mm, 64 × 64 matrix; 30 slices, voxel size = 3.75 × 3.75 × 4 mm). In each run, 220 scans were acquired. Additionally, 4 dummy scans were acquired to allow for steady magnetization. In a rapid event-related design, 138 trials (96 experimental trials + 32 null events) were presented at a rate of 4.5 s.

### Analysis

One participant was excluded from the analysis because of an overall error rate of 33%. For the remaining participants, reaction time (RT) as well as imaging analysis was based on correct trials only resulting in a loss of 8.4% of the data. Furthermore, response latencies falling outside the interval between 200 ms and 3500 ms were not considered and in a second step responses outside the interval of +/- 3 standard deviations around the individual mean were excluded. An additional amount of 0.26% of the data was excluded due to this trimming procedure.

In an initial analysis reaction times (RT) and error rates (ER) were analyzed using a 2 × 2 × 2 within-participant repeated measures ANOVA with the factors carry (carry vs. non-carry), problem size (small vs. large) and distractor type (distance between distractor and correct result 10 or 2). Furthermore, to pursue our main interest on the nature of the carry effect, a stepwise multiple regression analysis on mean item RT was conducted, which was stopped when inclusion of another predictor would not increase R^2 ^significantly (at *p *< .05). Replicating the analysis by [14] the predictors incorporated were *decade sum*, *unit sum, distractor type *as well as *carry-over*. The two predictors decade sum and unit sum simply reflect the sum of the digits at the decade or unit position of the two addends, respectively (i.e., ranging from 3, e.g., 31 + 12, to 17, e.g., 29 + 18). Contrarily, the predictors carry-over and distractor type were coded categorically: +1 in case the addition problem required a carry/the distractor differed by 10 from the correct result and -1 for problems not requiring a carry/the distractor differing by 2 from the correct result.

The anatomical scans were normalized and averaged in SPM8 [26]. The fMRI time series was corrected for movement artifacts and unwarped in SPM8. Images were motion corrected and realigned to each participant's first image. Data were normalized into standard stereotaxic MNI (Montreal Neurological Institute) coordinates space. Images were resampled every 3.75 mm using trilinear interpolation and smoothed with a 7.5 mm FWHM Gaussian kernel to accommodate inter-subject variation in brain anatomy and to increase signal-to-noise ratio in the images. The data were high-pass filtered (128 s) to remove low-frequency signal drifts and corrected for autocorrelation assuming an AR(1) process. Brain activity was convolved over all experimental trials with the canonical haemodynamic response function (HRF).

In analogy to the behavioural data, in all analyses only correctly answered trials were analyzed. The onsets of incorrect answered trials were entered separately as a condition of no interest into the models.

After carrying out a 2 × 2 × 2 within-participant repeated measures ANOVA analogous to the behavioral data, the effects of parametric predictors representing decade sum, unit sum, distractor type, and carry-over were estimated in a parametric analysis on the brain signal for each participant analogous to the regression analysis for the behavioral data (cf. [18]). In a second-level analysis, cortical regions showing modulation of signal specifically due to the parametric regressors were evaluated. Please note that the parametric analysis of brain signal is very fine-grained, so differences in brain signal explained by the respective predictors are given at a p-value of .005, uncorrected (except for the predictor decade sum).

The two predictors decade sum and unit sum simply reflect the sum of the digits at the decade or unit position of the two addends, respectively. For instance, the values for the predictor unit sum ranged from 3 to 17 (e.g., for 28 + 49, the sum of the unit digits would be larger: 8 + 9 = 17, while for 31 + 52, the sum of the unit digits would be smaller: 1 + 2 = 3). Thereby, when employed as a parametric predictor, one can either examine the activation which correlates with the increasing values of this vector (i.e., activation which increases with increasing values of the sum of the unit digits, e.g., from 3 to 17 in unit sum); or one can examine activation, which correlates with decreasing values of the vector (i.e., activation which increases the smaller unit sum gets, e.g., from 17 to 3 in unit sum).

For the anatomical localisation of the effects, we used the SPM Anatomy Toolbox [27], available with all published cytoarchitectonic maps from http://www.fz-juelich.de/ime/spm_anatomy_toolbox). This toolbox allows assessing the percent overlap of the fMRI activation with a cytoarchitectonic area as well as the position of the local maxima relative to all cytoarchitectonic areas. These maps provide information about the location and variability of cortical regions in a standard reference space (the MNI space). For areas not yet incorporated in this toolbox, we used the anatomical automatic labelling tool (AAL) in SPM8 [28]. The fMRI results were rendered on the surface of the right and left hemisphere of the SPM8 single subject template brain.

## Results

### Behavioural data

Before evaluating the properties of the carry effect, an initial ANOVA on the RT data served as a manipulation check to ensure the validity of the later, more specific analyses. Most important for the further evaluation of the carry effect, we observed that non-carry problems were easier than carry problems (cf. [14], and [29] for identical results on the same stimulus set). Additionally, items with large problem size were more difficult than items with small problem size. Thus, the prominent effects of carry and problem size were present in the current data set and warrant a closer inspection of the properties of these effects. For a full description of the ANOVA results the interested reader is referred to Appendix B.

### Continuity of the carry effect

The final model of the stepwise multiple regression incorporated the predictors decade sum, unit sum, and distractor type (R^2 ^= .70, adjusted R^2 ^= .69, *F*(3, 92) = 72.5, *p *< .001; see Table 1). The categorical predictor carry, which is highly correlated with unit sum, failed to explain a significant amount of additional variance. Inspection of the beta weights revealed that RT increased as the sum of the decade digits increased. More interestingly, RT also increased continuously as the sum of the unit digits of the addends increased; irrespective of the need for a carry-over. This again indicated (see also [14]) that the carry effect seemed determined by the added magnitudes of the unit digits, rather than being a purely categorical effect. The positive beta weight of the predictor distractor type indicated that probes with an incorrect decade digit (+/- 10) were *harder *to reject than probes with an incorrect unit digit (+/- 2).

**Table 1:** Outcome of the regression analyses: The table depicts the model specifications of a stepwise multiple regression model in which the four predictors decade sum, unit sum, carry, distractor type were incorporated.

	Regression weight				
Predictor	raw	standardized	*t*	**Change in R**^**2**^	*p*
Decade sum	119.87	.79	13.65	.54	< .001
Unit sum	43.98	.30	5.21	.10	< .001
Distractor type	119.30	.24	4.22	.06	< .001
*Carry-over*		*.16*	*1.48*		*.14*

The predictor carry (italics) was not considered in the final model.

### Imaging data

**Figure 1:**
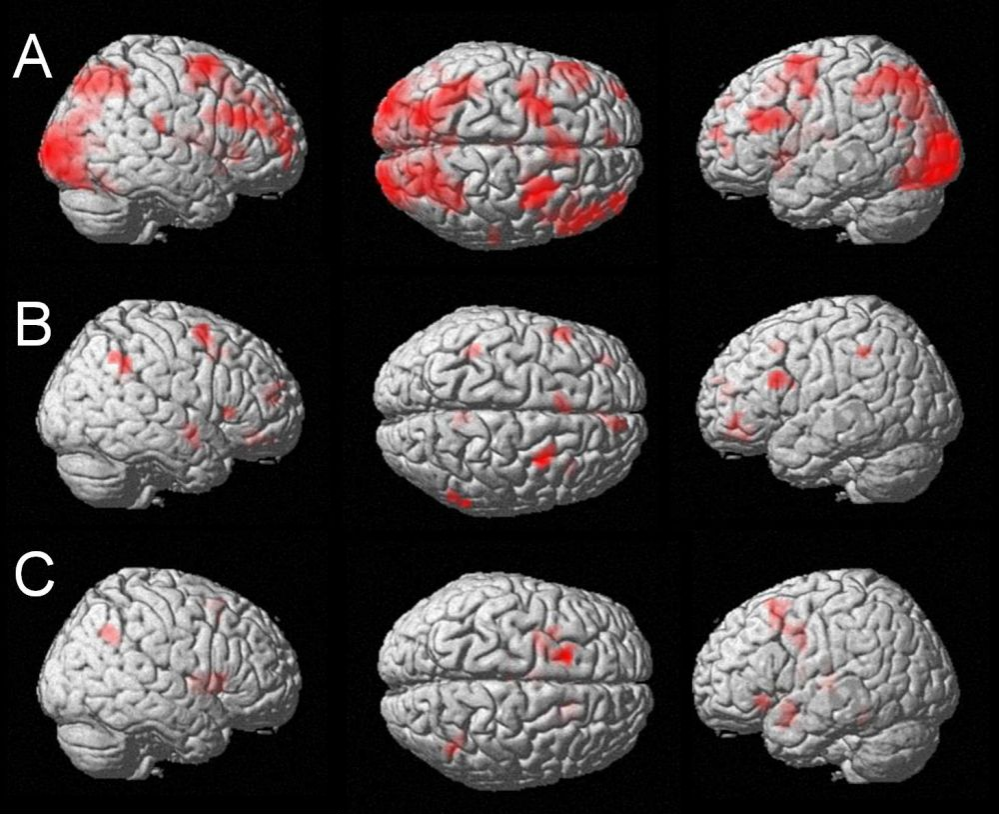
**ANOVA results**. A: Main effect of problem size at an uncorrected voxelwise p-value of < .00001 and cluster size k = 10 voxels: Bilateral intraparietal activation as well as activation of the left angular gyrus and the left retrosplenial cortex. Please note that a stronger p-value of < .00001 had to be used to enable a dissociation of the different maxima of activation (at a p-value of < .005 a cluster of > 10000 voxels covered large parts of bilateral occipital and parietal cortices). B: Main effect of carry at an uncorrected voxelwise p-value of < .005 and cluster size k = 10 voxels: Bilateral intraparietal activation as well as activation of the left angular gyrus and the left retrosplenial cortex. C: Interaction of problem size and carry: In the most difficult condition (i.e., large addition problems with a carry) right intraparietal activation is observed at an uncorrected voxelwise p-value of < .005 and cluster size k = 10 voxels.

Before evaluating the properties of the carry effect in a parametric analysis, an initial 2 × 2 × 2 ANOVA comprising the factors problem size, carry, and distractor type on the fMRI data revealed significant main effects of problem size and carry, which both included magnitude-related bilateral intraparietal activation (BA 7). Moreover, the factors problem size and carry interacted significantly. In the most difficult condition (i.e., carry addition problems with large problem size) a network of activation including the right intraparietal cortex (BA 7) was observed. This problem size by carry interaction was not present in the behavioural data of the current study, but was reported in previous behavioural studies [e.g., [5,30]]. Therefore, the ANOVA provides an interesting example for the case that neuro-imaging data can valuably complement behavioural data. The detailed ANOVA results are reported in Table 2 and Figure 1.

**Table 2:** ANOVA: Main effects of problem size and carry and their interaction.

Factor	Brain region (BA)	MNI (x, y, z)			Cluster size	F value
Problem size	LH posterior intraparietal sulcus (BA 7)	-26	-50	52	186	92.06
*Main effect*	RH posterior intraparietal sulcus (BA 7)	17	-67	52	149	71.09
	LH inferior frontal gyrus (BA 45)	-54	21	24	137	52.28
	RH inferior frontal gyrus (BA 45)	52	32	24	238	52.19
	RH inferior frontal gyrus (BA 47)	31	28	-1	24	40.25
	LH supplementary motor area (BA 6)	-5	14	48	315	74.46
	LH thalamus	-8	-18	10	341	51.32
	RH caudate nucleus	10	14	-12	11	33.99
	LH angular gyrus (BA 39)	-50	-63	24	13	29.53
	RH supramarginal gyrus (BA 40)	62	-28	20	19	32.09
	LH retrosplenial cortex (BA 31)	-5	-60	24	16	36.21
	LH middle frontal gyrus (BA 10)	-36	60	13	17	32.45
	RH middle frontal gyrus (BA 6)	31	4	59	155	82.04
	LH superior medial gyrus (BA 9)	-5	53	34	11	31.37
	RH inferior occipital gyrus (BA 18)	24	-98	-1	2209	98.98
						
Carry	LH intraparietal sulcus (BA 7)	-40	-39	45	10	17.64
*Main effect*	RH intraparietal cortex (BA 7)	59	-56	41	10	15.14
	LH inferior frontal gyrus (BA 44)	-54	14	24	21	19.37
	RH supramarginal gyrus (BA 40)	62	-46	38	10	20.43
	RH insula	38	21	6	11	18.38
	RH retrosplenial cortex (BA 31)	6	-49	31	18	15.75
	LH supplementary motor area (BA 6)	-5	18	45	14	24.05
	RH middle frontal gyrus (BA 6)	31	7	55	28	20.66
	RH superior medial gyrus (BA 32)	10	53	17	21	18.46
	RH rectal gyrus (BA 12)	3	39	-15	31	23.24
	LH middle orbital gyrus (BA 10)	-33	46	-5	10	17.72
	RH amygdala	27	-4	-8	31	23.34
						
Carry × problem size	RH intraparietal sulcus (BA 7)	45	-60	41	17	21.05
*Interaction*	LH insula	-29	25	-8	13	26.15
	RH pallidum	20	0	3	72	20.83
	LH thalamus	-15	-21	3	38	15.78
	LH superior frontal gyrus (BA 6)	-19	14	52	39	35.59
	LH precentral gyrus (BA 6)	-33	7	41	39	21.73

*p < .00001, p < .005, uncorrected; cluster size = 10 voxels; MNI: Montreal Neurological Institute coordinates.

Analogous to the regression analysis for the behavioral data, the effects of the parametric predictors representing decade sum, unit sum, distractor type, and carry-over were estimated in a parametric analysis on the brain signal.

#### Carry operation (categorical)

**Figure 2:**
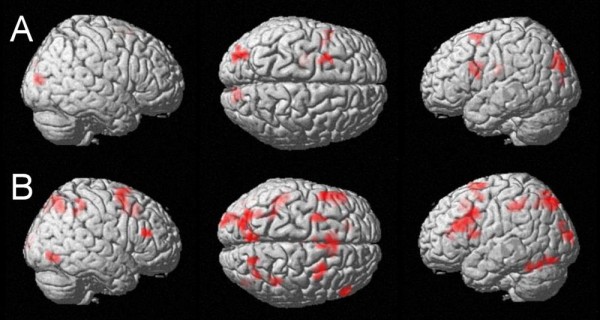
**Modulation of the fMRI signal by categorical and continuous properties of the carry effect**. A: fMRI activation significantly modulated by the requirement of a carry operation at an uncorrected voxelwise p-value of p < .005 and k = 10 voxels: please note the lack of significant magnitude-related activation. B: Cortical regions showing modulation of fMRI signal due to increasing unit sum (uncorrected p-value of < .005, cluster size k = 10 voxels): magnitude-related activation in the bilateral IPS.

The fMRI signal level was predicted by the presence of a carry operation (uncorrected p-value < .005, k = 10 voxels) in the left caudate nucleus (Figure 2A, Table 3), the left inferior frontal gyrus (BA 44, Broca's Area), the left middle frontal gyrus (BA 6), and the bilateral visual occipital cortices (BA 17 and BA 18).

**Table 3:** Cortical regions showing modulation of fMRI signal due to the effect of a carry operation or increasing unit sum.

Predictor	Brain region (BA)	MNI (x, y, z)			Cluster size	z value
Carry	LH caudate nucleus	-19	-15	19	11	3.25
	LH inferior frontal gyrus (BA 44)	-45	4	26	14	3.67
	LH middle frontal gyrus (BA 6)	-26	8	60	13	3.57
	RH calcarine gyrus (BA 17)	15	-83	8	20	4.44
	LH middle occipital gyrus (BA 18)	-30	-83	38	27	3.45
						
Increasing unit sum	RH posterior intraparietal sulcus (BA 7)	19	-68	56	15	3.93
	LH posterior intraparietal sulcus (BA 7)	-30	-71	53	20	3.53
	LH intraparietal sulcus (BA 7)	-34	-45	49	26	3.85
	RH intraparietal sulcus (BA 7)	41	-41	53	15	3.61
	LH precuneus (BA 7)	-4	-71	56	66	3.66
	LH fusiform gyrus (BA 37)	-49	-53	-23	14	4.35
	LH fusiform gyrus (BA 19)	-38	-71	-15	23	3.62
	LH inferior frontal gyrus (BA 44)	-56	11	11	26	3.94
	RH inferior frontal gyrus (BA 45)	53	34	19	11	3.69
	LH middle frontal gyrus (BA 9)	-53	11	38	76	3.92
	LH thalamus	-4	-11	11	13	4.01
	LH supplementary motor area (BA 6)	0	19	45	44	4.07
	LH supplementary motor area (BA 6)	0	11	68	19	3.72
	LH anterior cingulate gyrus (BA 32)	-19	38	11	11	4.12
	RH middle frontal gyrus (BA 6)	26	8	49	29	3.48
	LH superior frontal gyrus (BA 6)	-26	4	56	19	3.22
	LH middle occipital gyrus (BA 18)	-26	-98	15	23	3.66
	RH inferior occipital gyrus (BA 19)	45	-71	-11	14	3.89

p < .005, uncorrected; cluster size = 10 voxels; MNI: Montreal Neurological Institute coordinates.

#### Increasing unit sum

The fMRI signal was modulated significantly by increasing unit sum in the bilateral intraparietal sulci (BA 7) and the bilateral posterior intraparietal sulci (BA 7) at an uncorrected p-value < .005 and for a cluster size of k = 10 (Figure 2B, Table 3). Further clusters of activated voxels were observed in the left precuneus (BA 7), the left fusiform gyrus (BA 19 and BA 37), the bilateral inferior frontal gyri (BA 44 and BA 45, Broca's Area), the left thalamus, the left anterior cingulate gyrus (BA 32), the left dorsolateral prefrontal cortex (BA 9), the left supplementary motor area (BA 6), and in the bilateral middle/superior frontal gyri (BA 6) as well as the bilateral occipital cortices (BA 18 and BA 19).

In order to distinguish the cortical regions in which the fMRI signal was significantly stronger predicted by unit sum than by the categorical requirement of a carry operation, the influence of both predictors was directly tested using a paired *t*-test.

#### Unit sum vs. carry operation

**Figure 3:**
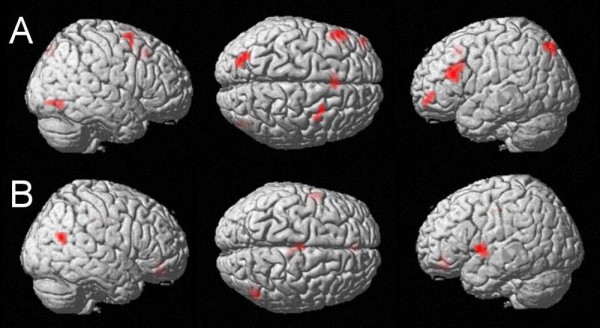
**Direct comparison of categorical vs. continuous properties of the carry effect**. A: Unit sum - carry at an uncorrected voxelwise p-value of p < .005 and k = 10 voxels. Magnitude-related activation in the left IPS, which is explained significantly better by the continuous predictor unit sum than by the categorical predictor carry. B: Carry - unit sum at an uncorrected voxelwise p-value of p < .005 and k = 10 voxels: Activation of the left Broca's Area as well as the right middle temporal gyrus bordering the angular gyrus.

Comparing cortical activations which are activated significantly stronger by unit sum than by carry operation at an uncorrected p < .005 and k = 10 voxels (Figure 3A, Table 4), we found activation in the left posterior intraparietal sulcus (BA 7), the left inferior frontal gyrus (BA 45, "Broca's Area") as well as in the left supplementary motor area (BA 6), the right middle frontal gyrus (BA 6), the right inferior occipital gyrus (BA 19), and the left middle orbital gyrus (BA 10).

**Table 4:** Cortical regions activated significantly more due to the predictor unit sum compared to the predictor carry and vice versa.

Contrast	Brain region (BA)	MNI (x, y, z)			Cluster size	z value
Unit sum vs. Carry	LH posterior intraparietal sulcus (BA 7)	-23	-79	45	19	3.17
	LH inferior frontal gyrus (BA 45)	-53	26	19	38	3.86
	LH supplementary motor area (BA 6)	-4	19	45	14	3.31
	RH middle frontal gyrus (BA 6)	30	8	56	11	3.60
	RH inferior occipital gyrus (BA 19)	41	-68	-8	11	3.43
	LH middle orbital gyrus (BA 10)	-45	53	-4	10	3.41
						
Carry vs. Unit sum	RH middle temporal gyrus (BA 39)	49	-64	19	12	4.01
	LH inferior frontal gyrus (BA 44)	-56	4	4	16	3.48
	LH middle orbital gyrus (BA 32)	-4	41	-11	18	3.28
	LH middle cingulate cortex (BA 24)	0	-19	41	11	3.28

p < .005, uncorrected; cluster size = 10 voxels; MNI: Montreal Neurological Institute coordinates.

#### Carry operation vs. unit sum

Contrasting activation predicted by carry operation to activation predicted by unit sum (Figure 3B, Table 4), significantly stronger activation was observed in the right middle temporal gyrus bordering the angular gyrus (BA 39), the left inferior frontal gyrus (BA 44, Broca's Area), the left middle orbital gyrus (BA 32), and the left middle cingulated gyrus (BA 24).

#### Increasing decade sum

**Figure 4:**
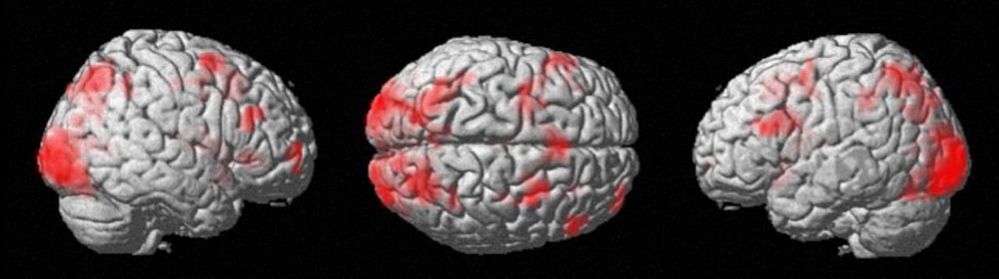
**Cortical regions modulated by increasing decade sum**. Magnitude-related activation in the bilateral IPS (uncorrected voxelwise p-value of < .00001 and cluster size k = 10 voxels).

The fMRI signal was modulated significantly by increasing decade sum in the bilateral posterior intraparietal sulcus (BA 7) and the left intraparietal sulcus (BA 7) at an uncorrected p < .00001 and cluster size of k = 10 (Figure 4, Table 5). Please note that for the determination of the coordinates as well as the generation of the figure a stronger p-value of < .00001 had to be used to enable a dissociation of the different maxima of activation. Further clusters of activated voxels were observed in the left inferior frontal gyrus (BA 44, "Broca's Area") extending into the dorsolateral prefrontal cortex (BA 9), the right inferior frontal gyrus (BA 44), the bilateral basal ganglia comprising the bilateral caudate nuclei, the left lentiform nucleus and the right thalamus. Increasing decade sum also modulated the fMRI signal in the left supplementary motor area (BA 6), the bilateral middle frontal gyri (BA 6 und BA 10), and in the visual occipital cortex (BA 18) extending into the fusiform gyrus (BA 37).

**Table 5:** Cortical regions showing modulation of fMRI signal due to further important factors in multi-digit addition.

Predictor	Brain region (BA)	MNI (x, y, z)			Cluster size	z value
Increasing decade sum*	RH posterior intraparietal sulcus (BA 7)	-24	-70	48	88	5.05
	LH posterior intraparietal sulcus (BA 7)	-30	-64	53	100	4.64
	LH horizontal intraparietal sulcus (BA 7)	-41	-41	38	155	4.85
	LH inferior frontal gyrus (BA 44)	-41	8	26	51	4.65
	RH inferior frontal gyrus (BA 45)	56	30	26	18	4.90
	LH caudate nucleus	-15	0	19	17	4.40
	RH caudate nucleus	11	11	-11	62	5.17
	RH thalamus	4	-19	11	18	4.24
	LH lentiform nucleus	-19	11	-11	11	4.00
	LH supplementary motor area (BA 6)	-4	19	45	62	5.07
	RH middle frontal gyrus (BA 10)	34	64	8	19	4.58
	RH middle frontal gyrus (BA 6)	30	8	60	42	4.63
	LH middle frontal gyrus (BA 6)	-26	-4	45	26	5.27
	LH fusiform gyrus (BA 37)	-38	-68	-19	19	4.38
	LH middle occipital gyrus (BA 18)	-26	-98	-4	871	5.75
						
Distractor type 10	RH intraparietal sulcus (BA 7)	45	-56	49	15	3.63
						
Distractor type 2	LH insula	-38	-15	15	16	3.54
	LH middle cingulate cortex (BA 23)	-8	-11	34	10	3.95

*p < .00001, uncorrected; p < .005, uncorrected; cluster size = 10 voxels; MNI: Montreal Neurological Institute coordinates.

#### Distractor type +/- 10

**Figure 5:**
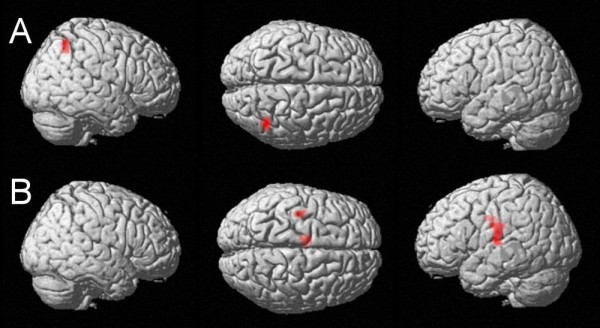
**fMRI signal modulations reflected by distractor type**. A: Cortical regions showing modulation of fMRI signal due to a distractor type of +/- 10 at an uncorrected voxelwise p-value of < .005 and cluster size k = 10 voxels: Magnitude-related activation in the right IPS. B: fMRI activation significantly modulated by the distractor type +/- 2: Activation of the left insula.

The brain region in which activity correlated with a distractor differing from the correct result by +/- 10 is depicted in Figure 5A and Table 5 (uncorrected p < .005, k = 5 voxels). The fMRI signal was modulated in the right intraparietal sulcus (BA 7).

#### Distractor type +/- 2

The fMRI signal was modulated by a distractor differing by +/- 2 from the correct result in the left insula and the left middle cingulated cortex (BA 23) at an uncorrected p < .005 and k = 5 voxels (Figure 5B, Table 5).

Finally, the use of a parametric analysis further enabled us not only to examine cortical sites in which fMRI signal correlated with increasing, but also with decreasing unit sum and/or magnitude of the addends involved. The two predictors decade sum and unit sum simply reflect the sum of the digits at the decade or unit position of the two addends (e.g., in 23 + 34 decade sum is 2 + 3 = 5 and unit sum 3 + 4 = 7). For instance, the values for the predictor unit sum ranged from 3 to 17 (e.g., for 28 + 49, the sum of the unit digits would be larger: 8 + 9 = 17, while for 31 + 52, the sum of the unit digits would be smaller: 1 + 2 = 3). Thereby, when employed as a parametric predictor, it is possible to examine both (i) the activation which correlates with the increasing values of this vector (i.e., activation which increases with increasing unit sum from 3 to 17) or (ii) the activation, which correlates with decreasing values of the vector (i.e., activation which increases the smaller unit sum gets from 17 to 3). We hypothesized that in addition problems with small problems (e.g., 4 + 3 = 7) and/or problems not requiring a carry operation (e.g., 12 + 15 = 17) cortical networks might be involved which are usually assumed to subserve simple fact retrieval (e.g., [20]).

#### Decreasing decade sum

**Figure 6:**
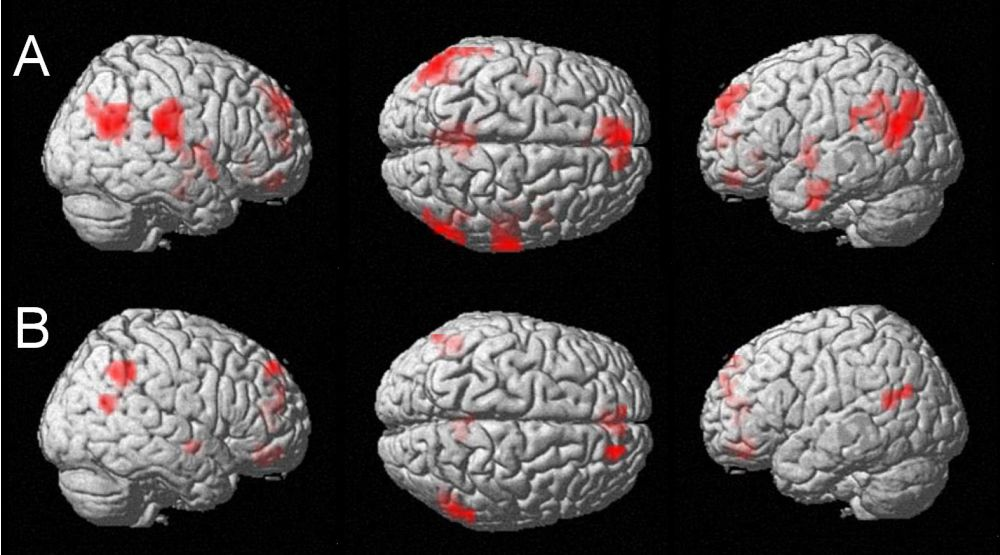
**Increasing fMRI signal changes with decreasing decade/unit sum**. A: fMRI signal change predicted by decreasing decade sum (uncorrected p < .005, k = 10 voxels) comprising the bilateral angular, the right supramarginal gyrus, the right hippocampus as well as the left retrosplenial cortex. B: fMRI activation significantly modulated by decreasing unit sum (uncorrected p-value of < .005, cluster size k = 10 voxels) included the left angular gyrus and the right retrosplenial cortex.

Cortical regions, in which the fMRI signal was predicted by decreasing decade sum were determined at an uncorrected p-value < .005 and cluster size of k = 10 (Figure 6A, Table 6). Interestingly, the fMRI signal correlated with decreasing decade sum in the bilateral angular gyri (BA 39), the right supramarginal gyrus (BA 40), the left inferior temporal gyrus (BA 21), the right putamen, the right hippocampus, the left retrosplenial cortex (BA 31), and the bilateral insula. Further modulated voxels were observed in the anterior cingulate gyrus (BA 23) and the left superior medial (BA 9) and the left rectal gyrus (BA 11).

**Table 6:** Cortical regions showing modulation of fMRI signal due to decreasing decade sum and decreasing unit sum.

Contrast	Brain region (BA)	MNI (x, y, z)			Cluster size	z score
Decreasing decade sum	LH angular gyrus (BA 39)	-45	-68	26	134	4.58
	RH angular gyrus (BA 39)	60	-56	34	78	4.08
	RH supramarginal gyrus (BA 40)	60	-26	23	112	4.34
	LH inferior temporal gyrus (BA 21)	-53	-8	-34	27	3.59
	RH putamen	30	0	4	21	3.57
	RH hippocampus	26	-11	-15	18	3.36
	LH insula	-41	-4	4	25	3.26
	RH insula	45	-4	4	19	3.07
	LH retrosplenial cortex (BA 31)	-4	-53	23	173	4.71
	LH anterior cingulate cortex (BA 23)	-11	34	0	18	3.64
	LH superior medial gyrus (BA 9)	-4	49	30	126	4.62
	LH rectal gyrus (BA 11)	-4	49	-15	33	3.96
						
Decreasing unit sum	LH angular gyrus (BA 39)	-53	-64	24	21	3.25
	RH middle temporal gyrus (BA 39)	53	-60	19	13	3.49
	RH supramarginal gyrus (BA 7)	56	-56	41	20	3.34
	RH retrosplenial cortex (BA 31)	8	-53	30	18	3.51
	RH putamen	30	-8	-8	11	3.29
	RH superior frontal gyrus (BA 9)	19	53	45	12	4.18
	RH middle orbital gyrus (BA 10)	8	53	-11	42	3.87
	LH superior medial gyrus (BA 9)	-8	53	26	38	3.69

p < .005, uncorrected; cluster size = 10 voxels; MNI: Montreal Neurological Institute coordinates.

#### Decreasing unit sum

fMRI signal change increased with decreasing unit sum at an uncorrected p-value < .005 and a cluster size of k = 10 (Figure 6B, Table 6) in the left angular gyrus (BA 39), the right supramarginal gyrus (BA 7), the right middle temporal gyrus (BA 39), the right retrosplenial cortex (BA 31), the right putamen, the right superior frontal gyrus (BA 9), the right orbital gyrus (BA 10), and the left superior medial gyrus (BA 9).

No modulation of the fMRI signal was predicted by the absence of a carry operation.

## Discussion

The current study set off to investigate properties of the carry effect and its neural correlates. We were primarily interested whether the behavioural findings from a previous study [14], suggesting both categorical and continuous aspects of the carry effect, could be extended to the neuro-functional processing level. In particular, we wanted to find out whether differentiation of processing characteristics into parietal magnitude-related as compared to (pre)frontal, more general processing demands would be feasible. In addition, two more issues will be addressed. First, we also evaluated the effects of two factors generally agreed upon to determine addition performance: magnitude of the addends (i.e., problem size) and the attributes of the distractors. Second, applying a parametric analysis to the present fMRI data enabled us to examine cortex sites for which the fMRI signal correlated not only with increasing unit sum (as one way to transfer RT regression factors to fMRI data) but also with decreasing unit sum and/or magnitude of the addends involved. Thereby, we were able to gain insights into additional processes underlying addition performance possibly related to the retrieval of arithmetical facts.

### The nature of the carry effect

Only recently, Klein et al. [14] observed that both categorical and continuous processing characteristics seemed to constitute the carry effect in addition. Our data corroborated this distinction at the behavioural level but also identified neural correlates of both aspects.

The behavioural data revealed that the carry effect is indeed not only purely categorical; rather, it seems to involve continuous properties reflected by the influence of the unit sum as well. In particular, the categorical factor carry was highly significant in the ANOVA for both RT and ER. However, unit sum was included as significant predictor in the final model of the regression analysis on item RT, whereas the categorical predictor carry was not considered. Thereby, the regression analysis suggested that response latencies increase commensurately with increasing unit sum indicating that the carry effect seems to be determined by this continuous factor as well.

Moreover, applying a parametric analysis to the neuro-functional data activation associated with processing either categorical and continuous aspects of the carry operation could be directly contrasted. This analysis revealed activation peaks in two distinct parts of the fronto-parietal network assumed to underlie number processing [16]. On the one hand, *continuous *aspects of the carry effect were associated with a different part of the network primarily involving the intraparietal sulcus (IPS) bilaterally. There is general agreement that the IPS is involved in processing numerical magnitude information [16] as well as in processes of place-value integration in multi-digit number processing [5,17,30]. However, previous data already indicated that the predictor unit sum may be highly correlated but not collinear with the categorical carry predictor, thus accounting for a specific part of the variance [14]. Therefore, the direct contrast of contributions of these two predictors on the BOLD response was of particular importance. While previous behavioural evidence only indicated that the effect of a carry operation can neither be regarded as exclusively categorical nor as exclusively continuous, our functional neuro-imaging data revealed that separate specific parts of a neural network contribute to the processing of categorical and continuous characteristics of the carry effect.

Moreover, the direct contrast of the continuous and the categorical predictor further specified the results described above. While brain activation for unit sum included areas in the IPS bilaterally and the posterior IPS, the direct comparison with the predictor carry only revealed posterior intraparietal activation. Generally, the involvement of bilateral IPS may indicate increased magnitude processing (e.g. [16,31]) with increasing unit sum. In contrast, the posterior intraparietal cortices have been discussed to be involved in the mental decomposition of the base-ten structure of digits (e.g., [18,30]), the activation of number sensitive and number selective coding systems [32], the mental manipulation of visuo-spatial information (e.g., [19,33], and mental imagery (e.g., [34])). In particular, the posterior IPS activation due to the predictor unit sum (19, -68, 56) is very close to the activation peak (21, -63, 57) that has recently been attributed to number sensitive coding systems [32], while the posterior IPS activation for the direct contrast unit sum vs. carry (-30, -71, 49) seems to be more number specific as it is rather related to the number selective coding system (-36, -60, 57). Thus, this posterior intraparietal activation may represent number selective integration of the spatially differentiated magnitudes of tens and units into the place-value system of the Arabic number system. And in particular, it may reflect the necessity to update and reevaluate place-value information as required in a carry operation (indicated by increasing unit sum) by moving the carry from the units to the tens slot.

On the other hand, the part of the network reflecting the *categorical *aspect of the carry effect was subserved by the basal ganglia as well as left-hemispheric language areas (Broca's Area). The basal ganglia have been associated with problem solving processes, cognitive set shifting, mental flexibility, and verbal memory (e.g., [35]), particularly involving phonological processing such as subserved by Broca's area. This pattern was further specified by the direct contrast of carry vs. unit sum. Most importantly, this contrast revealed no specific activation in bilateral magnitude-related cortical areas around the intraparietal sulcus. Instead, left-hemispheric language areas (Broca's Area) and the right middle temporal gyrus bordering the angular gyrus were observed to be active. Specifically, the activation observed in Broca's area may indicate that in carry addition problems the carry-over has to be kept in mind (cf. [36]) - being either required or not in an all or none manner.

Taken together, the present data corroborate the notion of the carry effect as being driven by both categorical and continuous aspects of numerical information [14]. However, our neuroimaging data even suggest a more specific assumption of concomitant processing of categorical and continuous characteristics of the carry effect subserved by different parts of a fronto-parietal network associated with numerical cognition (see [16] for a review).

### Factors determining addition performance

Further factors determining addition performance, such as the magnitude of the addends involved, and attributes of the distractors used were also examined in the present study. The effect of problem size was highly significant in the ANOVA on both RT and ER, with addition problems being more difficult when involving relatively larger addends. Moreover, decade sum was a significant predictor in the final model of the regression analysis. More importantly, in line with previous studies investigating activation due to the magnitude of the addends involved (e.g., [20]), an increasing sum of the decade digits resulted in the activation of a large fronto-parietal network. This network comprised the horizontal segment of the IPS (hIPS), which has repeatedly been identified to be vitally involved in processing number magnitude (for reviews [16,31]). Furthermore, the medial wall of the bilateral posterior IPS was involved, which has not only been associated with visuo-spatial processing as well as place-value integration [30,37], but also more recently with number sensitive (but not selective) coding [32]. Additionally, also the precuneus was found active, which has been suggested to contribute to quantity representation [38] as well as mental imagery and working memory [39,40]. Moreover, also the involvement of the left dorsolateral prefrontal cortex may indicate increased working memory demands and additional operations in calculation (e.g., [12,41,42]), but also processes of cognitive control (e.g., [13]). Furthermore, activation in Broca's area additionally seems to point to verbal working memory with the DLPFC and the inferior frontal gyrus both forming the neural correlate of inner rehearsal processes while performing problems with relatively larger decade sums. Finally, we observed increased occipital activation extending into the left fusiform gyrus, where the visual identification area for digit strings is supposed to be located [36].

Taken together, the current data corroborate previous findings indicating that addition performance is not only determined by the carry effect but also by the magnitude of the addends. The larger the addends, the higher are the demands on processing magnitude information as well as on working memory processes, and processes of visual identification.

On the other hand, also attributes of the distractors used in the present choice reaction paradigm were relevant. The factor distractor type not only proved to be significant in the ANOVA on both RT and ER (indicating that addition problems presented with a distractor differing at the unit position were easier to reject than distractors differing at the tens position), but was also incorporated in the final model of the regression analysis. Even more interestingly, the question whether the distractor differed from the correct result in tens or units position was associated with different patterns of neural activation: When the distractor differed from the correct result in decade position, magnitude-related IPS activation was observed, whereas a difference in unit position resulted in language-related activation of the left insula. Generally, this finding suggests decomposed processing of tens and units as discussed in more detail in the following.

### Decomposed processing of tens and units

Both, the finding of the carry effect being driven by the unit sum as well as the impact of the distractor differing at the ten's position are relevant regarding the notion of decomposed processing of tens and units [15,43,44]. In this view, the magnitudes of tens and units constituting two-digit numbers are represented separately. To activate the overall magnitude of a two-digit number, the single-digit magnitudes of tens and units need to be assigned their respective value and subsequently to be integrated into the base-10 place-value structure of the Arabic number system. In the case of two-digit addition, increased unit-decade integration demands occur whenever a carry operation is needed or whenever the distractor offered differs from the correct result at the tens position. As in the case of a carry operation, the sum of the unit digits is equal to or larger than 10, the decade digit of the unit sum has to be carried to the tens position to yield the correct result. A similar problem occurs in the case of a distractor, which differs from the correct result at the tens position, because the problem has to be calculated to the end (including the computation of the correct decade digit) to decide between the two alternative results provided. Taken together, the current data indicate that in choice-reaction of two-digit addition problems both, the carry effect as well as the effect of distractor type, are accounted for by an integration of decomposed representations of tens and units into the place-value structure of the Arabic number system. The concept of a decomposed representation of two-digit numbers was already invoked for the case of number comparison [44] as well as two-digit addition [14]. However, the present study provides first empirical evidence that decomposed processing of tens and units also activates a magnitude-related network when applied to mental arithmetic, because it accounts for both the effect of unit sum as well as the effect of distractor type.

To sum up, processes of unit-decade integration may index a particular case of processing a number's magnitude [14,30]. The significant magnitude-related IPS activation supported the following interpretation. Apart from the effect of the addend's magnitude, also the effects of distractor type and carry effect can be associated with number magnitude processing.

### Evidence for fact retrieval in addition problems

The use of a parametric analysis enabled us to also examine cortex sites in which the fMRI signal correlated with decreasing unit sum and/or magnitude of the addends involved. In addition problems with small addends (represented by a small decade sum) as well as in problems with small unit sum, cortical networks were observed which are usually assumed to subserve simple fact retrieval (e.g., [20]) as well as storage and retrieval of semantic knowledge as recently put forward in a meta-analysis [45]. In particular, both decreasing decade sum as well as decreasing unit sum was associated with fMRI signal change in the left angular gyrus. As also observed in previous studies (e.g., [46,47]), the increased fMRI brain signal change in the left angular gyrus due to decreasing decade sum and unit sum indicated a less strong degree of underactivation (see [45] for a more detailed discussion of this point). However, generally, the left angular gyrus has been associated frequently with the storage and retrieval of rote verbal representations of arithmetical facts as assumed for multiplication or addition with small numbers (possibly below 10; [16,48-52]) and, more recently, also with a rather general semantic system for efficient retrieval and manipulation of semantic knowledge [45].

Nevertheless, multi-digit addition is supposed to be a mixed operation, where online magnitude manipulation, procedural rules, and fact retrieval are assumed to be involved [53]. Combined with the notion of decomposed processing of tens and units and the fact that the increased fMRI signal change for the left angular gyrus was present for both decreasing decade and decreasing unit sum the following interpretation stands to reason. Our results indicate that access to arithmetic fact retrieval may not be limited to overlearned arithmetic facts (2 + 3 = 5) involving small (single)-digit addends whereas multi-digit problems may be solved by magnitude manipulations. Rather, these data imply that for addends with as small decade or unit sum arithmetical fact knowledge for single-digit additions smaller than 10 (e.g., 2 + 3 = 5) may be recruited for column-wise processing of the respective problems (cf. [36]). Thereby, the overlearned solutions to single-digit additions can be recycled to contribute to the overall solution of multi-digit problems (see [44] for a more detailed discussion). In sum, the involvement of the left angular gyrus in these tasks might reflect that participants split a more complex calculation into problems requiring retrieval of the simple arithmetic facts.

Such an interpretation is in line with the observed increased bilateral fMRI signal change in the supramarginal gyri. Goebel et al. [54,55] suggested that the supramarginal gyrus may also contribute to rote verbal fact retrieval. Additionally, increased fMRI signal change was also observed in retrosplenial cortex due to both, decreasing decade and decreasing unit sum. Again, this fits with an interpretation suggesting the involvement of fact retrieval processes when solving multi-digit addition problems. The retrosplenial cortex has been implicated in the recognition of familiarity (e.g., [56]). When assuming that arithmetic facts of small operand additions are highly overlearned, these should also be more familiar - thus, again suggesting that participants may have recruited arithmetic fact knowledge to solve problems with small decade sum and/or small unit sum.

Taken together, as previously observed for multiplication facts (cf., [47,49]), mental addition seems to rely (at least partially) on the recruitment of rote verbal arithmetic facts. This is consistent with the notion of arithmetic fact retrieval being involved not only in trained multiplication facts, but also in small over-learned addition problems. However, the present data extend the notion of fact retrieval to the case of larger addition problems whenever either decade or unit sum of the problem is small. These problems may be solved by breaking them down into simpler (over-learned) problems, most likely by column-wise processing.

### Limitations

Generally, it should be noted that intraparietal activation has also been associated with processes related to response selection/execution and task difficulty. For instance, Goebel and colleagues [19] have shown that response selection and number processing activate overlapping areas in the IPS. Transferred to the current study this means that IPS activation associated with increasing decade and/or unit sum could be driven by a general task component such as response selection or task difficulty rather than exclusively reflecting quantitative processing of number magnitude information. Therefore, we cannot exclude that IPS activation for increasing unit sum or increasing decade sum might be partly due to response selection demands. While most studies investigating the effect of number magnitude on brain activations have not been able to exclude these alternative explanations, Cappelletti et al. [57], aimed at dissociating IPS activation due to response selection from magnitude-related activation by partialling out effects of response time before evaluating activation differences between numerical and non-numerical conditions. Also Klein et al. [14] used both passive tasks and mental active tasks to systematically evaluate IPS activation which is not due to response selection or execution. Nevertheless, it has to be noted that in the contrast which is actually at the heart of the study - the direct contrast between the predictors carry and increasing unit sum - comparable effects of response selection as well as task difficulty should have occurred in both data sets. Thus, the corresponding activation patterns associated with response selection should have cancelled out each other when compared directly.

## Conclusions

The current study addressed three main issues. First, we were able to replicate the impact of problem size and distractor attributes on determining addition performance. Second, our findings suggest that arithmetic fact retrieval may not only be involved in small overlearned addition problems, but also in case of multi-digit addition problems. Finally and most importantly, we were interested in the properties of the carry effect (i.e., categorical and/or continuous) and its neural correlates. We observed two distinct parts of the fronto-parietal network dedicated to numerical cognition to be associated with either one of these two characteristics. On the one hand, the categorical aspect of the carry effect (need for a carry or not) was subserved by left-hemispheric language areas as well as the basal ganglia probably reflecting increased demands on procedural and problem solving processes. On the other hand, the continuous aspect of the carry effect was associated with increased intraparietal activation possibly indicating both increasing demands on magnitude processing as well as unit-decade integration when a unit sum increases and a carry operation becomes necessary.

In sum, representations and processes underlying the carry effect and their interplay seem to be more complex than assumed previously. In either case, exploring the representations underlying the carry effect is a promising way to learn more about the nature of multi-digit arithmetic.

## Competing interests

The authors declare that they have no competing interests.

## Authors' contributions

KW, HCN, FD and GW conceived the study. All authors participated in its design. KD and EK performed data collection, processing and the statistical analyses. EK and KM drafted the manuscript; the other authors revised it critically. All authors contributed to the interpretation of the data. All authors read and approved the final manuscript.

## Appendix A

### ANOVA for behavioural data

Main effects were present for problem size [RT: *F*(1,15) = 290.11; *p *< .001; ER: *F*(1,15) = 4.79; *p *< .01], carry-over [RT: *F*(1,15) = 45.95; *p *< .001; ER: *F*(1,15) = 4.79; *p *< .05], and distractor type [RT: *F*(1,15) = 19.08; *p *= .01; ER: *F*(1,15) = 12.13; *p *< .01]. Problems with a large problem size were responded to slower and more error prone than problems with a small problem size (2112 ms vs. 1864 ms and 11% vs. 6%, respectively), non-carry problems were responded to faster and less error prone (1855 ms and 6%, respectively) than carry problems (2102 ms and 10%, respectively) and problems with a distractor of +/- 2 size faster and more correctly than with a distractor of +/- 10 (1846 ms vs. 2112 ms and 6% vs. 10%, respectively). A two-way interaction of problem size and distractor type for reaction times [RT: *F*(1,15) = 18.10; *p *< .01; ER: *F*(1,15) < 1] indicated that the problem size effect was more pronounced for problems with distractor type +/- 10 (1011 ms) as compared to problems with distractor type +/- 2 (562 ms).

Moreover, there was a significant three-way interaction of distractor type, problem size, and carry [RT: *F*(1, 12) = 4.82, *p *< .05; RT; ER: *F*(1, 12) = 10.17, *p *< .01]. Breaking down this interaction into its constituting two-way interactions revealed that the interaction of carry and problem size was significant only for the distractor type +/- 10 [RT: *F*(1,15) = 6.86; *p *< .05; ER: F(1,15) = 5.07; p < .05], but not for the distractor type +/- 2 [RT: F(1,15) <1; p = .36; ER: F(1,15) = 4.05; p = .06]. This indicated that the effect of a carry operation was only larger in large problems (312 ms and 8%, respectively) than in small problems (116 ms and -2%, respectively) when the distractor differed from the correct result at the decade digit, but not at the unit digit.
